# Infection Preventionist and Hospital Epidemiologist Staffing in Acute Care Hospitals: Results from the PITAS Study

**DOI:** 10.1017/ash.2025.377

**Published:** 2025-09-24

**Authors:** Monika Pogorzelska-Maziarz, Elizabeth Monsees, Pamela De Cordova, Lucas Philip, Patrick Moeller, Tara Schmidt, Lauren Satchell, Patricia Stone

**Affiliations:** 1 Villanova University; 2 Children’s Mercy Hospital; 3 Rutgers, the State University of New Jersey; 4 Columbia School of Nursing

## Abstract

**Background:** Despite the crucial roles Infection Preventionists (IPs) and Hospital Epidemiologists (HEs) have in the implementation of patient safety strategies, there is paucity of data on what constitutes effective IP and HE staffing. Disparities in staffing and resource allocation in IPC and HE departments are currently underexplored. This study aims to evaluate staffing patterns, resource allocation, and collaboration among IPs and HEs comparing multi-facility organizations to free standing hospitals. **Methods:** All IPC departments in hospitals participating in the National Healthcare Safety Network were invited to participate in an electronic survey between August and December 2023. Data were collected on hospital and IPC department characteristics including organizational structure, IP staffing and resources, as well as HE staffing and time allocation. Descriptive statistics were used to summarize the data; Wilcoxon rank-sum tests and chi-square tests were used to compare variables between single, free-standing and multi-facility hospitals. **Results:** Responses were received from 901 IPC departments representing 2779 NHSN facilities. The majority of respondents were situated in multi-facility (61%) vs. free-standing hospitals (39%). Over half (57%) of the IPC programs were located under Quality (65% vs. 45% for multi-facility and free-standing hospitals, respectively) and 18% under Nursing (12% vs 27% for multi-facility and free-standing hospitals, respectively). Compared to free-standing hospitals, ICP departments in multi-facility hospitals reported higher median number of IPs (3.0 vs.1.0; p) **Conclusion:** This study reveals differences in IPC staffing and resources between free-standing and multi-facility hospitals with free-standing hospitals facing notable challenges, including limited access to HEs/ID Physicians. Future research should focus on identifying optimal staffing models and resource allocation to address these staffing and resource disparities and ensure equitable access to expertise. Interventions that leverage the collective expertise between IPC and HE are necessary to advance patient safety.

